# Detection of identity by descent using next-generation whole genome sequencing data

**DOI:** 10.1186/1471-2105-13-121

**Published:** 2012-06-06

**Authors:** Shu-Yi Su, Jay Kasberger, Sergio Baranzini, William Byerley, Wilson Liao, Jorge Oksenberg, Elliott Sherr, Eric Jorgenson

**Affiliations:** 1 Ernest Gallo Clinic and Research Center, University of California San Francisco, 5858 Horton St. Suite 200, Emeryville, CA, 94608, USA; 2 Department of Neurology, University of California San Francisco, 5858 Horton St. Suite 200, Emeryville, CA, 94608, USA; 3 Department of Psychiatry, University of California San Francisco, 5858 Horton St. Suite 200, Emeryville, CA, 94608, USA; 4 Department of Dermatology, University of California San Francisco, 5858 Horton St. Suite 200, Emeryville, CA, 94608, USA

## Abstract

**Background:**

Identity by descent (IBD) has played a fundamental role in the discovery of genetic loci underlying human diseases. Both pedigree-based and population-based linkage analyses rely on estimating recent IBD, and evidence of ancient IBD can be used to detect population structure in genetic association studies. Various methods for detecting IBD, including those implemented in the soft- ware programs fastIBD and GERMLINE, have been developed in the past several years using population genotype data from microarray platforms. Now, next-generation DNA sequencing data is becoming increasingly available, enabling the comprehensive analysis of genomes, in- cluding identifying rare variants. These sequencing data may provide an opportunity to detect IBD with higher resolution than previously possible, potentially enabling the detection of disease causing loci that were previously undetectable with sparser genetic data.

**Results:**

Here, we investigate how different levels of variant coverage in sequencing and microarray genotype data influences the resolution at which IBD can be detected. This includes microarray genotype data from the WTCCC study, denser genotype data from the HapMap Project, low coverage sequencing data from the 1000 Genomes Project, and deep coverage complete genome data from our own projects. With high power (78%), we can detect segments of length 0.4 cM or larger using fastIBD and GERMLINE in sequencing data. This compares to similar power to detect segments of length 1.0 cM or higher with microarray genotype data. We find that GERMLINE has slightly higher power than fastIBD for detecting IBD segments using sequencing data, but also has a much higher false positive rate.

**Conclusion:**

We further quantify the effect of variant density, conditional on genetic map length, on the power to resolve IBD segments. These investigations into IBD resolution may help guide the design of future next generation sequencing studies that utilize IBD, including family-based association studies, association studies in admixed populations, and homozygosity mapping studies.

## Background

The concept of identity by descent (IBD), which is used to indicate when alleles at a given locus in two individuals are inherited from a common ancestor, has played a fundamental role in many genetic studies. Analyses of IBD are commonly used in pedigree data for linkage mapping [1]. IBD also has many uses in population-based studies, including mapping disease genes [2,3], estimating haplotypic phase [4] and inferring evolutionary history (*e.g.*, natural selection and inbreeding depression) [5,6]. More recently, IBD has been applied to analyzing gene expression in related or unrelated individuals [7]. Incorporating such information about shared genetic material between individuals in linkage/association analyses has been shown to improve statistical power for mapping disease genes in some studies [8-10].

The length of an IBD segment will depend on the number of generations between the individ- uals under study and their common ancestor, as IBD tracts are broken down by recombination events over time. In family data, the common ancestor is fairly recent, and thus IBD segments are expected to be long. Long IBD segments are easily detected with low density genotype data; in fact, linkage analysis studies were typically conducted with 300–400 highly polymorphic mi- crosatellite markers, prior to the widespread use of microarray-based genotyping. This approach takes advantage of the fact that tracts of IBD extend several centiMorgans (cM) across family members, covering many genetic variants. On the other hand, the expected length of IBD seg- ments between two putatively unrelated subjects in a large population is expected to be small. In this case, deeper coverage of genetic variants obtained through sequencing can increase the power to detect small IBD segments. As whole genome sequence data becomes increasingly avail- able, quantifying the extent to which sequence data can improve the resolution of IBD detection is an important step toward enabling more powerful approaches to disease gene mapping and understanding population history.

Various methods for detecting IBD have been proposed for use with population genotype data, including methods based on observed long segments of allele sharing [4,11] and on probabilities of IBD built into a hidden Markov model (HMM) (Pur- cell *et al.*, 2007; [2,12-15]). We focus our attention on two high computationally efficient methods implemented in the software packages, Germline [11] and fastIBD [14]. The former searches for IBD by directly matching portions of haplotypes between individuals from phased genotype data. The later detects IBD segments by modeling shared haplotype frequencies accounting for background levels of LD based on an HMM from unphased genotype data. In addition to the computational efficiency, fastIBD accounts for un-certainty of haplotype phase while inferring IBD states. Previous studies using high-density SNP genotype data from European ancestry samples in the HapMap project have shown that these two programs have good power to detect IBD segments greater than 2 cM in length [14].

Here, we conduct a comprehensive evaluation of IBD detection using sequence data, and compare the resolution of detectable IBD segment lengths with that of microarray-based genotype data using these two software packages (Germline and fastIBD). We investigate the power and false positive rate of these two approaches for microarray genotype data from the WTCCC study, denser genotype data from the HapMap Project, low coverage sequencing data from the 1000 Genomes Project, and deep coverage complete genome data from our own projects. The results of our analysis can help guide the design of future next generation sequencing studies that utilize IBD.

## Results

**Figure 1:**
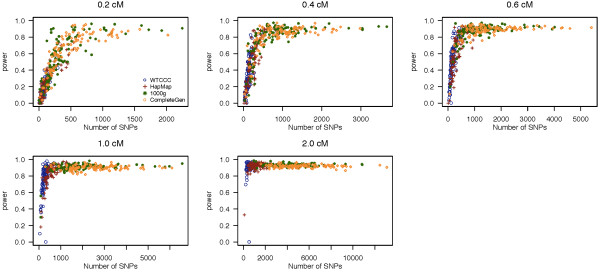
**Empirical power of fastIBD.** Empirical power of fastIBD to detect an IBD segment as a function of the number of SNPs within a segment in the simulation study. Each plot presents different lengths of IBD segments examined. The power of each dataset is represented by different colored circles and plotted against the number of SNPs contained within a given region.

In Figure 1, we present the power to detect a segment of IBD as a function of the number of SNPs within that segment for the five IBD segment lengths examined in this study using the program fastIBD. The average power across 100 simulated datasets is shown in Table 1. Using sequence data with high density SNP coverage improves the power to resolve IBD segments significantly over microarray-based genotype data, particularly for small IBD segments. The fastIBD method is able to detect IBD segments 0.2 cM in length with a power of 62.9% for high coverage sequence data (Complete Genomics), while it only has power of 12.6% for low density microarray genotype data (WTCCC). Power increases both as a function of the length of the IBD segment examined and the number of SNPs in the segment. Sequence data provides greater numbers of SNP genotypes for the same segment length than microarray-based genotype data. For all four datasets, fastIBD provides good power 76.7% to detect IBD for segments of size 1 cM and larger.

**Table 1:** The average power of fastIBD and Germline

	fastIBD				Germline			
Segment Size (cM)	WTCCC	HapMap	1000 g	complete	WTCCC	HapMap	1000 g	complete
0.2	0.126	0.251	0.562	0.629	0.043	0.344	0.665	0.645
0.4	0.327	0.518	0.781	0.801	0.099	0.551	0.836	0.806
0.6	0.495	0.649	0.864	0.874	0.135	0.617	0.901	0.907
1	0.767	0.840	0.904	0.899	0.231	0.794	0.941	0.944
2	0.909	0.918	0.935	0.919	0.389	0.905	0.982	0.992

**Figure 2:**
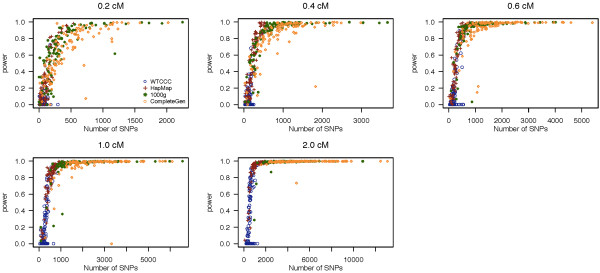
**Empirical power of Germline.** Empirical power of Germline to detect an IBD segment as a function of the number of SNPs within a segment in the simulation study. Each plot presents different lengths of IBD segments examined. The power of each dataset is represented by different colored circles and plotted against the number of SNPs contained within a given region.

In Figure 2, we present the power to detect a segment of IBD of as a function of the number of SNPs within that segment for the five IBD segment lengths examined in this study using Germline, and Table 1 shows the average power across 100 simulated datasets. We observed a similar trend between the power and number of SNPs and the examined IBD segment sizes, where Germline reports 66.5% power to detect IBD segments of size 0.2 cM. However, Germline has poor power with low density microarray genotype data (from WTCCC), even for detecting large IBD segments of size 2 cM. Comparing Germline with fastIBD, fastIBD has greater power for low density genotype data than Germline, whereas Germline has slightly higher power for sequence data.

**Figure 3:**
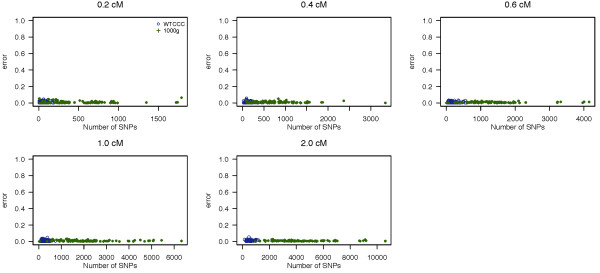
**Empirical false positive rate of fastIBD.** Empirical false positive rate of fastIBD to detect an IBD segment as a function of the number of SNPs within a segment in the simulation study. Each plot presents different lengths of IBD segments examined. The error rate of each dataset is represented by different colored circles and plotted against the number of SNPs contained within a given region.

**Figure 4:**
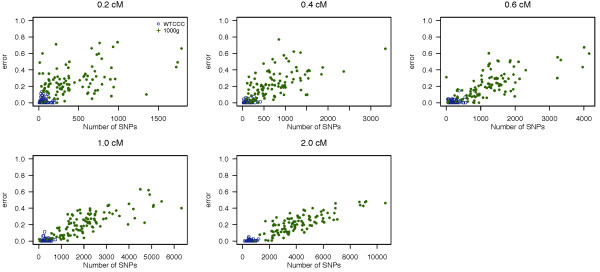
**Empirical false positive rate of Germline.** Empirical false positive rate of Germline to detect an IBD segment as a function of the number of SNPs within a segment in the simulation study. Each plot presents different lengths of IBD segments examined. The error rate of each dataset is represented by different colored circles and plotted against the number of SNPs contained within a given region.

The false positive rate (FPR) using fastIBD and Germline are presented in Figures 3 and 4, respectively, as a function of the number of SNPs within a segment. We summarize the average FPR across 100 datasets in Table 2. The FPR of fastIBD is low (<= 0.011) for both low and high variant density datasets (WTCCC and 1000 Genomes projects), and lower than that of Germline for sequence data (1000 Genomes data). We observed that Germline has fairly high FPR for sequence data, which increases linearly with the number of SNPs within a segment.

**Table 2:** The average false positive rate of fastIBD and Germline

	fastIBD		Germline	
Segment Size (cM)	WTCCC	1000 g	WTCCC	1000 g
0.2	0.009	0.009	0.015	0.269
0.4	0.007	0.009	0.006	0.239
0.6	0.008	0.007	0.009	0.225
1	0.010	0.009	0.006	0.223
2	0.011	0.009	0.003	0.218

**Figure 5:**
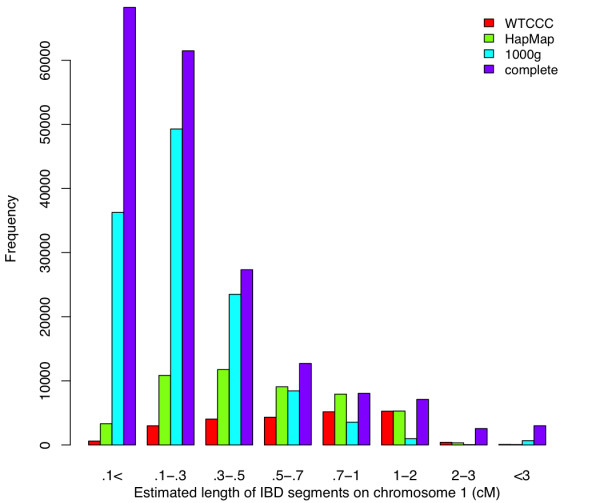
**The distribution of detected lengths of IBD segments across four investigated datasets using fastIBD.** We report the counts of detected IBD segments against estimated segment lengths on Chromo- some 1 for 60 individuals in each of the datasets (54 individuals for the deep coverage sequencing dataset).

Finally, we determined the background distribution of IBD between subjects using all SNPs on Chromosome 1 from each of the four empirical datasets. fastIBD provides the estimated length of an IBD segment along the chromosome, and we estimated IBD for all possible pairs of 60 individuals (54 individuals for deep coverage sequence data) for each of the four datasets. We present the distribution of IBD segment lengths in Figure 5. We normalized the results to adjust for the different numbers of individuals between datasets to make the results comparable. The deep coverage frequencies are multiplied by the ratio of (60 choose 2) to (54 choose 2). Overall, the total length of IBD segments detected on Chromosome 1 in the high variant coverage sequence data is longer than that in the low variant coverage data (Table 3), suggesting not just greater resolution but also greater detection of IBD using sequence data. This may be due to the fact that fastIBD is able to detect many more small segments of size 0.5 cM or smaller with the use of sequence data (high variant density) than that using the low density genotype dataset.

**Table 3:** The total length (cM) of IBD segments detected on Chro- mosome 1 using fastIBD

	WTCCC	HapMap	1000g	complete
number of individuals	60	60	60	54
total length (cM)	17409	26843	29372	46936

## Discussion

In this study, we examined how the density of genetic variants in a dataset affects the power to detect IBD between individuals. We found that analysis of sequence data with high SNP density improves resolution and power for detecting IBD relative to microarray-based genotyping, particularly for small segments. In our simulation, there was good power (80%) to detect IBD segments of size 0.4 cM using high coverage sequence data with a low false positive rate, compared to a power of approximately (77%) for segments of size 1 cM using microarray genotype data (WTCCC).

It is possible that the methods we examined in this study may be further refined to improve the power to detect even smaller IBD segments. We found that Germline has slightly higher power to detect IBD using sequence data compared to fastIBD, but it has a much higher false positive rate. That is, for high variant density data, Germline detects many small segments, where around 25% of them are false positives. We set the detectable minimum length to 0.1 cM while running Germline, which allows Germline be able to detect small segments, but it increases the false positive rate. Germline also provided lower power for detecting IBD segments using the microarray dataset (from WTCCC). These results indicate that fastIBD provides more robust and reliable IBD detection than Germline for these types of datasets. Given these observations, the current implementation of fastIBD appears to be better than the current implementation of Germline for detecting IBD segments for both low and high variant density data. We note that fastIBD represents a recent update to the HMM approach to IBD detection [2,3,13,14], and was previously tested on microarray data.

The results of this study have important implications for the design of genetic studies of human diseases. Identity by descent estimation can be used to conduct family-based association studies, association studies in admixed populations, and homozygosity mapping, and improved resolution and detection of IBD can enhance the power of these approaches to detect human disease genes. In general, the expected length of IBD is 1/(2n) Morgans for a common ancestor from n generations ago for a large population, where the ancestral haplotype is transmitted across 2n meioses. The variance of the length of an IBD track, however, is large, and the expected IBD lengths in a relatively small population could be affected dramatically by some aspects of population history (*e.g.*, growth type and internal subdivision) [16]. In fact, recent studies have shown that both the amount of the genome shared identical by descent and the proportion of the genome that is covered by long runs of homozygosity differs by population [5,17,18]. A more detailed assessment of IBD across populations could help determine to what extent whole genome sequence data can improve the power of these mapping approaches. Additionally, the continued improvement of IBD detection methods and the testing of those methods on dense genetic data can provide a foundation for future genetic studies.

## Materials and methods

### Data

To assess the statistical power to detect chromosomal segments that are shared identical by descent between two individuals, we conducted a simulation study. First, we collected genotype data from four sources that represent different levels of coverage (that is, the proportion of all variants in the genome that are assayed by a given platform), ranging from microarray genotype data to deep coverage whole genome sequence data. These empirical genotype data include: Microarray genotype data (WTCCC) from the Wellcome Trust Case Control Consortium (WTCCC) study. We obtained genotype data on 1000 controls from UK National Blood Donors (NBS) cohort genotyped on the Illumina 1.2 M chip. We used the SNP set released from the WTCCC database, which represents a cleaned set of data from their default QC procedures [19]

Denser genotype data (HapMap) from the HapMap phase II project. We obtained genotype data on 60 unrelated samples from the CEU population (Utah residents with ancestry from northern and western Europe) Low coverage sequence data (1000 g) from the 1000 Genomes Project. We obtained genotype data on 283 individuals that originate from Europe sequenced with 4X coverage (2010.08 release).

Deep coverage sequence data (complete) from University of California at San Francisco Whole Genome Sequencing Consortium and Complete Genomics [20]. We obtained genotype data on 54 samples of European origin sequenced by Complete Genomics with an average of 50X coverage. We used the Complete Genomics default cut offs for full genotype calls (excluding partial and no calls), which pass a strict quality score metric.

### Construction of artificial IBD for assessing power

**Figure 6:**
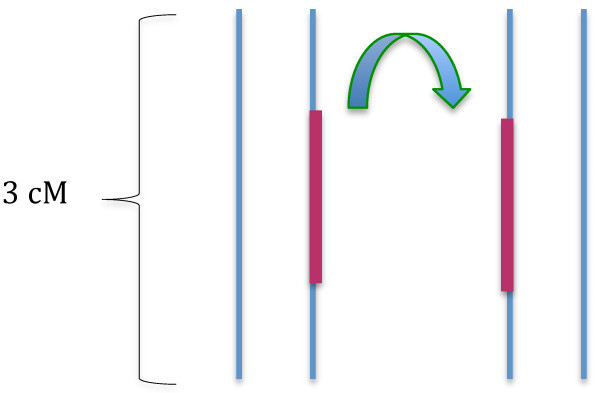
**Illustration of the procedure for creating artificial IBD seg- ments.** For a pair of individuals, a segment of the chromosome (shown in red) within a randomly chosen region on Chromosome 1 is copied to one chromosome in another individual.

Next, to investigate the power to detect IBD of various lengths, we constructed artificial IBD segments by copying a haplotype from a chromosome of one individual into the same location in the other individual (Figure 6). We simulated 30 pairs for WTCCC, HapMap and 1000 g data as well as 27 pairs for the deep coverage sequencing data, where individuals contain these artificial IBD segments for each of the datasets (a subset of individuals from the WTCCC and 1000 Genomes projects were selected). To assure that our results were not influenced by the structure of a single region, we randomly selected 100 regions, each 3 cM in length, on Chromosome 1, and conducted the simulation for each of these regions separately. In each region, we created artificial IBD segments of lengths 0.2, 0.4, 0.6, 1, and 2 cM. The program may detect several small segments within an artificially created IBD segment. Thus, we calculated the number of SNPs on these small IBD segments identified by the program. For each length of IBD segment, power was then assessed by taking the average proportion of SNPs that are detected as IBD within the simulated segments, over 100 regions and 30 pairs of individuals.

**Figure 7:**
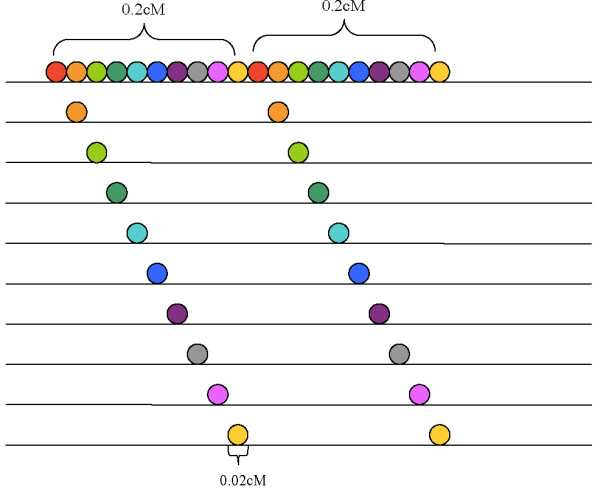
**Illustration of the construction of composite segments.** Each line represents the chromosome sequence of an individual. The colored circle represents the consecutive sequence of a segment size 0.02 cM, which may contain multiple SNPs. A composite segment of size 0.2 cM is composed of 10 consecutive segments of size 0.02 cM from 10 different individuals. To create a composite segment of size 0.4 cM, two composite segments of size 0.2 cM are constructed and merged. A similar procedure is conducted to create composite segments of size 0.6, 1 and 2 cM, where three, five and ten small composite segments are constructed and merged respectively.

Construction of composite individuals for assessing the false positive rate. We then investigated the rate of falsely detecting IBD when no IBD is present through a sec- ond simulation. We started by constructing a composite chromosomal segment for each of 10 simulated individuals [14]. To do this, we selected 100 individuals from the each of the WTCCC and 1000 Genomes datasets. Composite chromosome segments of length 0.2 cM were constructed by copying 10 consecutive regions of length 0.02 cM from 10 different individuals (Figure 7). To create segments of 0.4, 0.6, 1, and 2 cM, we combined 2, 3, 5, and 10 consecutive composite segments. In this way, we generated 10 simulated individuals with specific chromosomal segments that are not IBD. In this simulation setting, we expect that any pair of these 10 individuals with composite chromosomal segments are unlikely to share any part of that segment longer than 0.02 cM. Thus, detecting an IBD segment longer than 0.02 cM among these individuals can be considered as a false positive.

As in the first simulation, we investigated 100 regions on Chromosome 1 for each of the 5 different lengths of composite segments (0.2, 0.4, 0.6, 1, and 2 cM). A subset of 100 individuals from WTCCC and the 1000 Genomes datasets were randomly selected to create the 10 composite individuals. We included the other 900 individuals in the WTCCC data and 183 individuals in the 1000 Genomes data for IBD analysis. We did not investigate the error rate for HapMap and Complete Genomic data due to the limited number of individuals available for study. For each IBD segment length, the false positive rate is calculated by the number of SNPs that are detected as IBD divided by the total number of SNPs within the simulated segment. The error rates were then averaged over 100 regions and any pair of these 10 individuals. For Germline, the input data need to be phased genotype data. Thus, we phased the data before running Germline using fastIBD. Both fastIBD and Germline generate a list of all pairwise IBD segments.

We ran the fastIBD function in Beagle V3.3.1 with default settings. fastIBD applied a score threshold when detecting IBD. The results in the previous study shows that a threshold of 10^−10^ gives good power to detect IBD and also keep the false discovery rate close to zero [14]. Here, we used the default threshold 10^−8^. We used default settings in Germline V1.5.0 except that we set the minimum length (−min m) to 0.1 cM. This allows Germline to have a chance of detecting small segments.

## Competing interests

The author(s) declare that they have no competing interests.

## Authors’ contributions

SYS carried out the analysis and wrote the initial draft of the paper. JK provided assistant on accessing and manipulating the datasets. SB, WB, WL, JO and ES provided sequence data and edited the paper. EJ instigated/oversaw the project and edited the paper. All authors read and approved the final manuscript.
